# Influence of Wild and Cultivated Environments on the Antioxidant and Medicinal Components of *Rhodiola sachalinensis* A. Boriss.

**DOI:** 10.3390/plants13243544

**Published:** 2024-12-19

**Authors:** Xin Wei, Qiu-Yang Chang, Yang Liu, He-Nian Hua, Ya-Nan Liu, Zhong-Hua Tang, Li-Qiang Mu

**Affiliations:** 1 School of Forestry, Northeast Forestry University, Harbin 150040, China; 17614882590@163.com (X.W.); changqiuyang99@126.com (Q.-Y.C.); hhn54553627@nefu.edu.cn (H.-N.H.); lynm@nefu.edu.cn (Y.-N.L.); 2 Engineering Research Center of Agricultural Microbiology Technology, Ministry of Education, Heilongjiang University, Harbin 150500, China; 2021103@hlju.edu.cn; 3 College of Chemistry, Chemical Engineering and Resource Utilization, Northeast Forestry University, Harbin 150040, China; tangzh@nefu.edu.cn

**Keywords:** *Rhodiola sachalinensis* A. Boriss., phytochemicals, antioxidant properties, primary metabolites, secondary metabolites, sustainable cultivation

## Abstract

*Rhodiola sachalinensis* A. Boriss., recognized for its significant medicinal potential, is increasingly threatened by overharvesting in wild habitats. This study aims to elucidate the phytochemical and pharmacological distinctions between wild and cultivated *R. sachalinensis* to support sustainable cultivation practices. Utilizing UPLC and GC-MS, we conducted a comprehensive analysis of primary metabolites (e.g., soluble sugars, amino acids) and secondary metabolites (e.g., phenolic compounds, flavonoids) in both root and aboveground tissues from wild and cultivated sources. Results revealed that habitat plays a critical role in metabolite composition, with wild *R. sachalinensis* roots showing notably higher antioxidant properties and concentrations of key secondary metabolites, including epigallocatechin, which are linked to the plant’s medicinal efficacy. Conversely, cultivated plants demonstrated elevated levels of primary metabolites, reflecting adaptation to nutrient-rich, soil-based growth environments. These findings underscore the pharmacological importance of environmental factors in optimizing *R. sachalinensis* for medicinal use and highlight essential considerations for its sustainable cultivation strategies.

## 1. Introduction

*Rhodiola sachalinensis* A. Boriss., commonly known as the alpine Rhodiola in northeast China, belongs to the Crassulaceae family and the *Rhodiola* genus [1]. It is a perennial herbaceous plant. *R. sachalinensis,* also known as the “Oriental Divine Herb”, is primarily found in North America, Europe, and the northwestern regions of Asia [2]. In China, it is mainly found in the Changbai Mountain area of Jilin Province, counties such as Shangzhi, Ning’an, and Hailin in Heilongjiang. It grows on mountain slopes with altitudes between 1600 and 2500 m, under forests, on rocky slopes, and in alpine tundra [3].

*R. sachalinensis* is also one of China’s renowned medicinal plants, known for its fatigue resistance, anti-aging properties, and ability to withstand severe cold, and is highly valued by the medical community [4,5]. A high-altitude herbaceous species renowned for its pharmacological properties has been extensively studied for its antioxidant, anti-fatigue, and anti-aging effects, largely attributed to its phenolic and flavonoid compounds [6,7,8]. Therefore, *R. sachalinensis* is famous as the “Eastern God Herb” for its ability. However, with the increasing market demand, concerns over the source of *R. sachalinensis* are growing [9,10]. A significant amount of wild resources have been plundered, and given the fragile ecology of the alpine tundra, the habitats of most wild populations have become fragmented [11]. The resources of wild *R. sachalinensis* are gradually diminishing, prompting people to consider the possibility of its artificial cultivation. Today, both wild and cultivated environment *R. sachalinensis* coexist in the market. The differences and comparisons between them have become a hot topic among consumers, researchers, and the industry [12].

High-altitude habitats exhibit more complex habitat characteristics, including stronger radiation, less oxygen, lower temperatures, and specific limitations in high-altitude areas, which are crucial for plant growth, development, and physiological metabolism [13,14]. There are many substances in plants that can contribute to plant resistance, such as flavonoids and polyphenols, and these substances can resist low temperatures, UV radiation, etc. It is the plant that can resist them thanks to the protection provided by these compounds [5,15]. These substances can resist low temperatures, UV radiation, drought, or diseases to function in response to environmental changes [16,17,18,19]. Soluble sugars and soluble proteins are also important substances in plant growth and development processes. When abiotic stress affects plants, soluble sugars not only serve as metabolic sources and structural substances but also as signals to various processes of plant growth and development [20,21]. In the face of adversity, changes in soluble sugars and soluble proteins in plants are observed [20,22]. Typically, conditions like salt stress and low temperatures lead to an increase in soluble sugar concentrations, while exposure to high light, especially UV-B radiation and heavy metals, may reduce soluble sugar levels [22,23,24,25]. This stress varies in response to changes in the environment and genotype [26,27]. Such alterations represent a dynamic aspect of plant defense mechanisms and constitute a crucial feature in a plant’s ability to adapt to environmental fluctuations [28]. Therefore, studying the differences in antioxidant and chemical components between wild and cultivated environments of *R. sachalinensis* in high-altitude areas is of practical significance.

To verify differences in chemical constituents between wild and cultivated environments and whether wild environment *R. sachalinensis* has a better therapeutic effect than cultivated environments. We first compared the morphology of wild and cultivated environment species, as morphological characteristics are often a more direct and effective way to understand plant components [29]. The chemical constituents were identified, and the antioxidant, anti-inflammatory, and anti-proliferative capacities in wild and cultivated were determined in this study. At the same time, to understand the changes in physiological and metabolic indicators between wild and cultivated environments under long-term altitude differences. We used GC-MS and HPLC for qualitative analysis to directly compare compositions of wild and cultivated environments. We can gain a better understanding of the environmental effects on wild and cultivated *R. sachalinensis* through changes in metabolite profiles. The antioxidants in vitro were evaluated to compare the pharmacological effects of wild and cultivated *R. sachalinensis*.

Our research has played a representative role in the development of high-altitude *R. sachalinensis* resources in the Heilongjiang region, and the altitude differences between wild and cultivated environments are also of special significance. Despite the availability of some biological and chemical data on *R. sachalinensis*, comprehensive comparisons of its chemical composition and pharmacological potential between wild and cultivated environments remain limited. Understanding these differences is essential, as it addresses whether cultivated *R. sachalinensis* can serve as a viable substitute for wild populations without compromising therapeutic efficacy. This study thus aims to bridge this knowledge gap by examining the primary and secondary metabolite profiles, as well as the antioxidant properties, of wild and cultivated *R. sachalinensis*.

## 2. Materials and Methods

### 2.1. Samples

In this study, wild and cultivated *R. sachalinensis* were used to compare growth, physiological parameters, and metabolic changes under different environmental conditions. Wild samples were collected from Changting Town, Hailin City, Heilongjiang Province (44°26′40.85″ N, 128°18′36.79″ E) at an altitude of 1600 m. Cultivated samples were obtained from Taipinggou Nursery (43°6′6.77″ N, 128°26′17.82″ E) at an altitude of 600 m, creating a 1000 m altitude difference between the sites. The primary visible difference was that cultivated samples had significantly larger rhizomes.

To minimize age-related variability, only specimens of similar developmental stages were selected. Plant size and other developmental indicators were used to approximate maturity levels, as precise age markers for *R. sachalinensis* are unavailable. This selection aimed to reduce age variability and focus on environmental influences. To ensure consistency in soil composition between wild and cultivated environments, the soil used in the cultivated environment was sourced from the same altitude as the wild habitat. This controlled approach minimizes soil composition as a variable, allowing for a clearer focus on the effects of environmental conditions, such as altitude, on *Rhodiola sachalinensis* growth and metabolic characteristics.

Root length was measured by cleaning, rinsing with distilled water, and drying the roots with absorbent paper. Using a digital caliper, measurements were taken from the root crown to the tip of the longest root. Each sample was measured three times, with the average length used for analysis.

We collected samples of *R. sachalinensis* from both cultivated and wild environments to investigate morphological, physiological, and metabolic differences. To streamline references, we denote cultivated root samples as CR and wild root samples as WR. Similarly, cultivated leaf samples are referred to as CL, and wild leaf samples as WL.

Sampling was conducted in July 2022. Cultivated plants were grown in an open field in preparation for large-scale cultivation. All samples were freeze-dried using an Alpha 1–2 LD Plus freeze-dryer and stored at −20 °C. Triplicate analyses were performed on both wild and cultivated samples in all assays. Each group of samples is repeated three times. It is worth noting that each group repeats from the same plant within each group (e.g., CR1 contains three root replicates of a strain of *R. sachalinensis*). The plants were identified as *Rhodiola sachalinensis* A. Boriss. by Professor Mu Li Qiang.

### 2.2. Measurement of Soluble Sugars and Soluble Proteins

The soluble sugar (SS) content was determined by the anthrone colorimetric method using distilled water as a control, and the absorbance values at a 630 nm wavelength were measured under a UV spectrophotometer [30].

The soluble protein (SP) content was determined by the Komas Brilliant Blue staining method, using 0.3 mL of distilled water plus 5 mL of Komas Brilliant Blue solution as a control to determine the absorbance value at 595 nm under a UV spectrophotometer [31].

### 2.3. Determination of Total Phenolic and Total Flavonoid Content

In the determination of total phenols, 0.2 g of the *R. sachalinensis* sample was weighed and cut into small pieces. Then, 5 mL of 70% methanol was added, and the mixture was heated in a water bath at 70 °C for 10 min. The solution was allowed to cool, then centrifuged at 12,000× *g* rpm for 10 min. The supernatant was collected and diluted to 100 mL with 70% methanol. Ten milliliters of the test solution was added, and then 5 mL of 10% Folin–Ciocalteu reagent was added, mixed thoroughly, and allowed to stand for 10 min. Then, 4 mL of 7.5% anhydrous sodium carbonate solution was added, diluted with water to a final volume of 25 mL, shaken well, and allowed stand for 40 min. The absorbance at 765 nm was measured using a UV spectrophotometer. Methanol was used as the reference blank, as it serves both as a solvent for dissolving plant extracts and as a consistent baseline for absorbance measurements. A calibration curve was constructed using gallic acid solutions at concentrations ranging from 1 mg/mL. The total phenolic content (TPC) was expressed as gallic acid equivalents (GAE), quantified as mg GAE/g of extract.

To determine the total flavonoid content, rutin was used as the standard to construct a calibration curve. A stock solution of rutin (200 µg/mL) was prepared and diluted to obtain standard solutions of varying concentrations. For each standard solution, 0.3 mL of 5% sodium nitrite (NaNO_2_) solution was added, mixed thoroughly, and incubated for 6 min. Subsequently, 0.3 mL of 10% aluminum chloride solution was added, followed by another 6 min incubation. Then, 4 mL of 4% sodium hydroxide (NaOH) solution was added, and the mixture was diluted to a final volume of 10 mL with distilled water. After standing for 5 min, the absorbance was measured at 510 nm using a UV spectrophotometer. For sample analysis, accurately weigh 0.2 g of the sample and add 50 mL of 60% ethanol. Heat the mixture in an 80 °C water bath for 30 min, then cool and centrifuge it at 12,000 rpm for 10 min. The supernatant was collected and diluted to 100 mL with 60% ethanol. Two milliliters of the extraction solution was transferred into a new test tube, then 0.3 mL of 5% sodium nitrite solution was added, and the solution was mixed well and allowed to stand for 10 min. Next, 0.3 mL of 10% aluminum chloride solution was added, mixed, and allowed to stand for another 10 min. Finally, 4 mL of 4% sodium hydroxide solution was added and allowed to stand for 5–6 min. The absorbance was measured at 510 nm using a UV spectrophotometer. The total flavonoid content was calculated using the rutin calibration curve and expressed as milligrams of rutin equivalent per gram of extract (mg RE/g). Ethanol was used as the blank to ensure consistency and accuracy throughout the assay [32].

### 2.4. Antioxidant Assays

#### 2.4.1. 1,1-Diphenyl-2-Picrylhydrazyl (DPPH) Radical-Scavenging Activity Assay

The DPPH radical scavenging activity of the extracts was determined using a method described by Jing et al. [33], with some modifications. In brief, extracts were dissolved in a 70% ethanol solution to prepare various concentrations, and 750 µL of a 0.1 mM ethanol solution of DPPH was mixed with various sample solutions (0–1 mg/mL). After incubation at room temperature for 30 min in the dark, absorbance was measured at 517 nm. Ethanol was used as the blank control, and ascorbic acid was used as the positive control. The absorbance of the DPPH solution is 0.8. The DPPH scavenging activity was evaluated according to the following equation:

$$\mathrm{DPPH}\;\mathrm{radical}\;\mathrm{scavenging}\;\mathrm{activity}\;(\text{\%})\;=\;\left[1-\frac{\left(A_{1}-A_{2}\right)}{A_{0}}\right]\times 100\%$$

where A_0_ is the absorbance of the ethanol solution by DPPH without extracts, A_1_ represents the absorbance of the ethanol solution by DPPH with tested extracts, and A_2_ is the absorbance of the sample solution alone (without DPPH), which corrects for any absorbance contributed by the sample itself.

#### 2.4.2. 2,2′-Azino-bis(3-ethylbenzothiazoline-6-sulfonic Acid) (ABTS) Radical-Scavenging Activity Assay

The 2,2′-Azino-bis(3-ethylbenzothiazoline-6-sulfonic acid) (ABTS) radical scavenging activity of the extracts was determined using the method described previously with some modifications [33]. The stock solution of the ABTS solution was prepared by reacting a 7.4 mM ABTS solution with 3.8 mM potassium persulfate and was then kept in the dark at ambient temperature for 16 h. For the assay, the ABTS solution was diluted with ethanol to an absorbance value of 0.7 ± 0.02 at 734 nm. The diluted ABTS solution (3.9 mL) was added to 100 µL samples (0–1 mg/mL), absorbance was measured at 734 nm after 30 min, and the ABTS radical scavenging activity was calculated using the same equation as that for the DPPH scavenging activity.

### 2.5. GC-MS Conditions

The process of extracting and conducting GC-MS analysis on metabolic products from the root segments adhered to previously established protocols [34]. Samples of *R. sachalinensis* roots and leaves (0.1 g each) were placed individually in centrifuge tubes. To each sample, 540 μL of methanol and 60 μL of the internal standard (L-2-chlorophenylalanine, 0.3 mg/mL) were added, followed by mixing and sonication for 30 min. Next, 600 μL of water and 300 μL of chloroform were added, and samples were sonicated for an additional 30 min. The tubes were then centrifuged at 14,000× *g* rpm for 10 min at 4 °C, and 700 μL of the supernatant was collected.

To re-dissolve the supernatant, 200 μL of methoxyamine pyridine solution (15 mg/mL) was added, followed by incubation at 37 °C for 90 min. Subsequently, 200 μL of BSTFA (N,O-Bis(trimethylsilyl)trifluoroacetamide containing 1% trimethylchlorosilane) and 40 μL of n-hexane were added, followed by vortexing for 2 min and derivatization at 70 °C for 60 min. After derivatization, samples were centrifuged, and the supernatant was collected for GC-MS analysis.

GC-MS analysis was performed using an Agilent 7890A autosampler coupled with an Agilent 5975C GC-MS system(Santa Clara, CA, USA). Separation was achieved with a non-polar DB-5 capillary column (30 m × 250 μm ID, Thermo Fisher Scientific Mississauga, ON, Canada) using helium as the carrier gas at a flow rate of 1.0 mL/min. The column temperature program was as follows: heating from 60 °C to 125 °C at 8 °C/min, then from 125 °C to 210 °C at 4 °C/min, and finally from 270 °C to 305 °C at 10 °C/min, with a 3 min hold at 305 °C. The injection port temperature was set to 260 °C, with an injection volume of 1 μL. The mass spectrometer operated at an ionization voltage of −70 V, with a mass scan range of m/z 50–600. Data collection began after a 5 min delay, with a scan speed of 20 spectra per second.

### 2.6. HPLC Conditions

For the high-performance liquid chromatography (HPLC) analysis, a gradient elution method was used with mobile phase A as water and mobile phase B as acetonitrile. Separation was achieved using an Agilent TC-C18 column (4.6 mm × 250 mm, 5 μM). The detection wavelength was set to 280 nm. The wavelength of 280 nm was chosen for detection because many phenolic compounds, flavonoids, and other aromatic metabolites commonly present in plant extracts absorb strongly at this wavelength. This wavelength provides optimal sensitivity for detecting these bioactive compounds, making it a standard choice for analyses involving polyphenolic and flavonoid contents in plant extracts [35]. The flow rate was maintained at 0.8 mL/min, and the column temperature was controlled at 35 °C. A sample volume of 20 μL was injected. The gradient elution program is shown in Table S1.

Control samples of salidroside, tyrosol, rosavin, rhodiosin, rhodionin, quercetin, and kaempferol were accurately weighed and placed in a 50 mL volumetric flask. Methanol was added to dissolve the compounds, and the solution was sonicated to ensure complete dissolution, yielding the standard solution. These standards were analyzed by HPLC to generate standard curves.

An accurately weighed 0.25 g sample was placed in a mortar, and a total of 1.5 mL of 70% methanol was added in batches while thoroughly grinding. The mixture was transferred to a centrifuge tube and sonicated at 40 °C for 30 min. After sonication, the mixture was filtered, and the filter residue was rinsed with an additional 1.5 mL of 70% methanol. The resulting mixture was sonicated again at 40 °C for 30 min. The sample was then centrifuged at 6000 g for 10 min, and the supernatant was collected.

The supernatants from both extraction steps were combined and evaporated to dryness. The dry residue was dissolved in 0.5 mL of 70% methanol, sonicated for 10 min, and centrifuged at 12,000× *g* for 10 min. The final supernatant was collected for HPLC analysis.

### 2.7. Data Processing and Statistical Analysis

The metabolome data discussed below are an average of 3 repetitions. Statistical analysis was performed through analysis of variance (ANOVA), followed by Duncan’s multiple range test (SPSS26.0, IBM, Armonk, NY, USA); *p* < 0.05 was statistically significant, and GraphPad Prism 9 was employed for graphing.

Normalized GC-MS data were obtained through SIMCA-P version 11.0 (Umetrics, Umea, Sweden) for principal component analysis and partial least squares discriminant analysis. The candidate metabolites were screened by a variable influence on projection (VIP), with a combination method of value greater than 1.0 as well as a *p*-value below 0.05.

## 3. Results

### 3.1. Comparison of Growth Differences Between Wild R. sachalinensis and Cultivated R. sachalinensis

**Figure 1:**
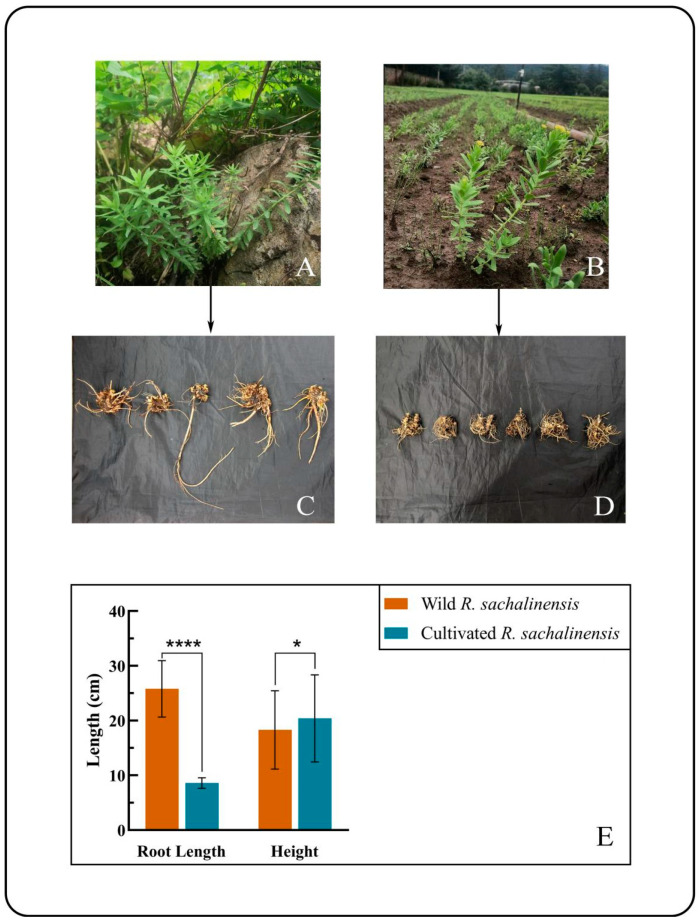
(**A**) Habitat of wild *R. sachalinensis*; (**B**) habitat of cultivated species; (**C**) root of wild *R. sachalinensis*; (**D**) root of cultivated *R. sachalinensis*; (**E**) comparison of root length and plant height between wild *R. sachalinensis* and cultivated species. “*” and “****” indicate statistical significance at *p* < 0.05 and *p* < 0.0001.

We compared the morphological differences, including the length of the underground part and the height of the aboveground part, between 30 wild *R. sachalinensis* plants and cultivated environments. As shown in Figure 1A,B from the survey data, we found significant differences in the aboveground parts between wild and cultivated types, as evident in Figure 1C,D. Notably, in the root system, there is a more substantial length difference between wild and cultivated types.

The average root length of wild *R. sachalinensis* is 25.8 cm, while the average root length of the cultivated species is 8.6 cm, showing a highly significant difference (Figure 1). Overall, the roots of wild *R. sachalinensis* are generally longer than those of cultivated environments. This difference could be attributed to the need for longer roots when these plants are naturally anchored to rocky surfaces, while the roots of cultivated environments are typically buried in the soil.

### 3.2. Comparison of Antioxidant Capacities and α-Glucosidase Inhibition Activity Between Wild and Cultivated R. sachalinensis

**Figure 2:**
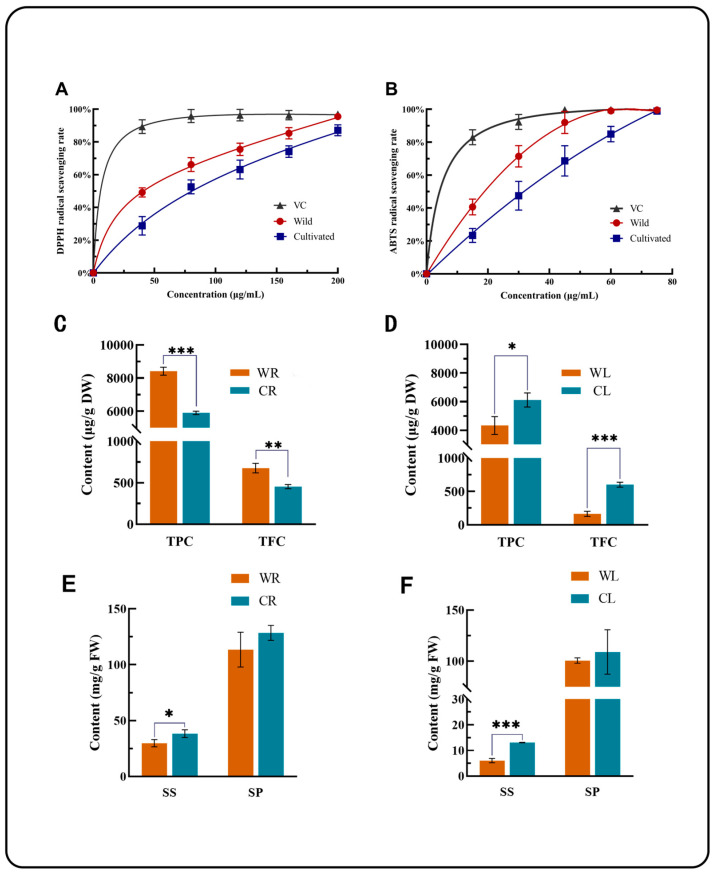
(**A**) DPPH radical scavenging activity assay radical scavenging activity results of WR and CR (**B**) ABTS radical scavenging activity assay scavenging activities results of WR and CR (**C**) The total flavonoid and phenolic contents in the WR and CR. (**D**) The total flavonoid and phenolic contents in the leaf of WR and CR. (**E**) Soluble sugar and soluble protein content in WR and CR. (**F**) Soluble sugar and soluble protein content in WL and CL. In Figure 2A,B, the abbreviation “VC” refers to vitamin C. It is used as a positive control in both the DPPH and ABTS radical scavenging assays to compare the antioxidant activities of the extracts with a well-known antioxidant. The symbols *, **, and *** indicate levels of statistical significance: * *p* < 0.05; ** *p* < 0.01; *** *p* < 0.001. These symbols represent significant differences between the antioxidant activities of the wild and cultivated samples at various concentrations. In all diagrams, the error bars represent the standard deviation (SD) of the mean values obtained from triplicate measurements. Each bar shows the variation around the mean, and the average values are reported in the text along with the standard deviation. This provides a measure of data reliability and variability within each sample group.

The antioxidant activities of the wild root (WR) and cultivated root (CR) extracts were assessed using DPPH and ABTS radical scavenging assays. As shown in Figure 2A, the DPPH radical scavenging activities of WR and CR extracts displayed a concentration-dependent trend. The WR extract demonstrated significantly higher scavenging activity compared to the CR extract. Specifically, the IC50 values for WR and CR were 45.34 μg/mL and 75.22 μg/mL, respectively, indicating that the WR extract had a stronger DPPH scavenging ability. At a concentration of 200 μg/mL, the scavenging rate of WR extract reached 95.5%, while the CR extract only achieved 87.06% (*p* < 0.05), confirming the superior antioxidant capacity of the WR extract in this assay.

Similarly, the ABTS radical scavenging activities of the two extracts, as shown in Figure 2B, also exhibited a concentration-dependent response. The WR extract showed greater scavenging effects on ABTS radicals compared to the CR extract at each tested concentration. The IC50 values were 18.39 μg/mL for WR and 28.38 μg/mL for CR, indicating that the WR extract is more effective in scavenging ABTS radicals. These findings suggest that the WR extract possesses higher overall antioxidant potential than the CR extract.

### 3.3. Difference in Total Flavonoid and Total Phenolic Contents Between Wild R. sachalinensis and Cultivated R. sachalinensis

The differences in total phenolic content (TPC) and total flavonoid content (TFC) between wild roots (WR) and cultivated roots (CR) were measured. As shown in Figure 2C, the results revealed that the TPC and TFC levels in WR were 8416.48 μg/g and 677.037 μg/g, respectively, while in CR, the TPC and TFC levels were 5903.25 μg/g and 453.58 μg/g, respectively. The TPC level in WR was significantly higher than that in CR (*p* < 0.001), and similarly, the TFC level showed a significant difference (*p* < 0.01). These findings align with the previously obtained antioxidant indices, indicating that WR contains higher antioxidant substances, such as flavonoids and phenolic compounds, compared to CR.

Interestingly, as illustrated in Figure 2D, the TPC and TFC levels in the leaves of the cultivated environment were higher than those in the wild environment. In WL, the TFC and TPC contents were 4331.07 μg/g and 161.28 μg/g, respectively, whereas in CL, these values were 6114.89 μg/g and 601.317 μg/g, respectively. The cultivated environment significantly surpassed the wild type (*p* < 0.01). This trend contrasts with the observed data in the roots.

### 3.4. Difference of Soluble Sugars and Soluble Proteins in R. sachalinensis

The content of SS (solid sugar) and SP (solid protein) in WR and CR is shown in Figure 2E, and both SS and SP have CR greater than WR. The content of SS and SP in CR is 38.34 mg/g and 128.49 mg/g, respectively, while the content in WR is 29.83 mg/g and 113.50 mg/g, respectively. The SS level of CR showed a significant difference compared to the SS level of WR (*p* < 0.05), while the SP content of CR did not show a significant difference compared to the SP content of WR. This result also occurred in CL and WL, where the SS content of CL showed a significant difference compared to WL (*p* < 0.001), while the SP content of CL did not show a significant difference compared to WL. The content of SS (solid sugar) and SP (solid protein) in WL and CL is shown in Figure 2F, and both SS and SP have CL greater than WL. The SS and SP levels in CL are 13.12 mg/g and 109.02 mg/g, respectively, while the content in WR is 6.04 mg/g and 100.64 mg/g, respectively.

### 3.5. Comprehensive Analysis of GC-MS Result Between Wild R. sachalinensis and Cultivated R. sachalinensis

**Figure 3:**
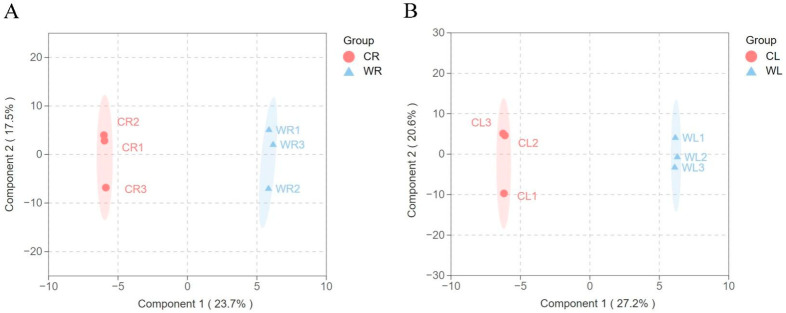
OPLS-DA score plot for wild and cultivated of *R. sachalinensis* ((**A**) roots; (**B**) leaves).

In order to study the metabolic differences between wild *R. sachalinensis* and cultivated *R. sachalinensis*, we first used the OPLS-DA model for analysis. This model is a regression method that models the relationship between multiple independent variables (X) and a categorical variable (Y) [36]. it distinguishes between orthogonal and non-orthogonal variables, enabling a more precise interpretation and separation. Through permutation testing, we obtained that the R2Y of these two models reached 0.989 and 0.985, and Q reached 0.57 and 0.75, respectively, which met the experimental expectations and met the analysis requirements. They can effectively explain the metabolic differences between wild and cultivated environments. The horizontal and vertical coordinates in OPLS-DA (Figure 3A,B) represent inter-group and intra-group differences, respectively. From the figure, it can be seen that there are significant differences in metabolites between wild and cultivated environments, both in the root and aboveground parts.

A total of 169 metabolites were detected in the leaves, and 180 metabolites were detected in the roots. These metabolites included acids, alcohols, ethers, aldehydes, ketones, amino acids, and sugars. To further narrow down differentially expressed metabolites, we applied the variable importance projection (VIP) in the orthogonal projections to latent structures discriminant analysis (OPLS-DA) model with a threshold of VIP > 1. Additionally, metabolites were selected based on *t*-test results with a significance level of *p* < 0.05, distinguishing between the roots and aboveground parts. A total of 10 differential metabolites were identified in the roots, comprising quinones (alizarin), alcohols (butanediol), phenolic compounds (epigallocatechin, phenol), sugars (galactopyranos, gentiobiose), and an acid (xylonic acid). In contrast, seven differential metabolites were found in the aboveground parts, including organic acids (citric acid, gallic acid, glyceric acid, and phosphoric acid), carbohydrates (fructose, galactose oxime, and sucrose), and fatty acids and alcohols (glyceric acid, stearic acid), as well as amino acids (serine, threonine, and valine).These findings provide valuable insights into the metabolic differences between the roots and aboveground parts of wild and cultivated environments, shedding light on the diverse array of metabolites in these plant tissues.

**Figure 4:**
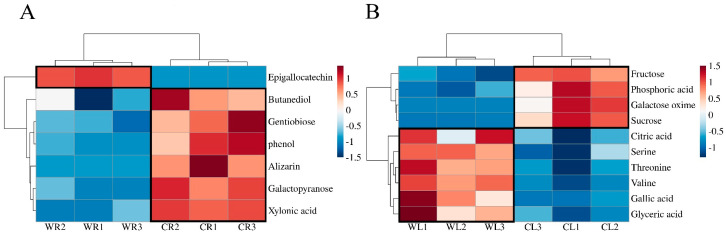
Heat maps showing significant changes in metabolites in the roots and aboveground parts of wild-type and cultivated types ((**A**) roots; (**B**) leaves).

To observe the changes in differential metabolites more intuitively, we used cluster heatmap analysis to compare the differences between wild and cultivated environments. (Figure 4A,B). In the face of environmental change, cultivated plants tend to accumulate sugars and other metabolic products. Notably, we found that in the roots of *R. sachalinensis*, only epigallocatechin was down-regulated, and the other differential metabolites were up-regulated. Epigallocatechin plays a unique role in plant–environment interaction, especially in the aspect of stress resistance, and the accumulation of epigallocatechin may be different in the face of different altitudes and other conditions [1] (Figure 4A). However, this phenomenon has changed in the aboveground part (Figure 4B). Fructose, phosphoric acid, galactose oxime and sucrose are up-regulated in the wild type, which is different from citric acid, serine, threonine, valine, gallic acid, and glycerol acid are up-regulated in the cultivated type, which indicates that compounds changed by environmental stress are common when altitude is the key factor affecting plant development.

### 3.6. Comprehensive Analysis of Bioactive Compounds in Two R. sachalinensis Environment

**Figure 5:**
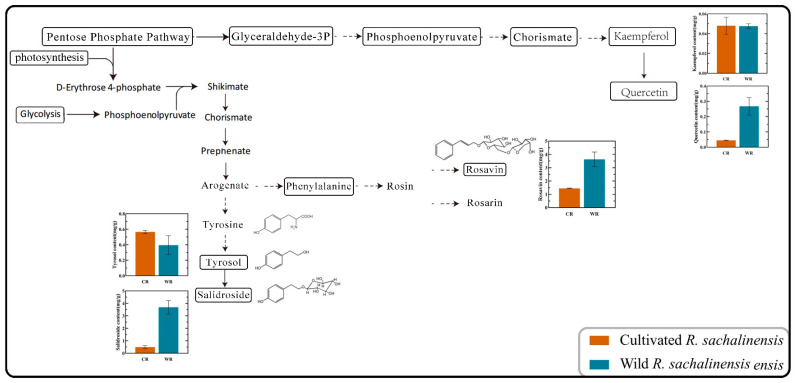
Schematic diagram of the biosynthetic pathways of the main medicinal components of CR and WR. The bar chart represents the content of this component in CR and WR (mg/g).

Although metabolomics can widely detect components, further research is needed on specific medicinal active ingredients. The main active ingredients in *Rhodiola* include salidroside, tyrosol, rosavin, rhodiosin, and rhodionin. These components mainly occur in the shikimate pathway, and tyrosol is the key to synthesizing salidroside [37]. In the secondary metabolites section, we determined seven noteworthy medicinal and active ingredients in WR and CR root with HPLC. Firstly, we observed the changes of five components during the synthesis process through the synthesis pathway (Figure 5). We found that the changes in these secondary metabolites in the pathway were characteristic, with Salidroside, the most active ingredient, far exceeding CR in WR by 3.68 mg/g and in CR by 0.48 mg/g. The key precursor substance of salidroside, tyrosol, has a higher content in CR than in WR, with a concentration of 0.39 mg/g in WR and 0.56 mg/g in CR. From the graph, we can see that arogenate mainly produces two branches: one mainly produces tyrosol, and the other mainly produces rosavin and rosarin. The content of rosavin in WR is 3.63 mg/g, while in CR it is 1.45 mg/g. Overall, the content in WR is higher; quercetin and kaempferol are also two important secondary metabolites of flavonoids, which have antioxidant and anti-inflammatory effects. The content of kaempferol in WR and CR is almost the same, which is 0.048 mg/g, while quercetin in WR is 0.27 mg/g, which is significantly higher than that in CR (0.04 mg/g). Rhodiosin and rhodionin are also the main active ingredients in rhodiola, and we compared these two components (Figure S1). The results showed that rhodiosin in WR was 1.2 times higher than in CR, while rhodionin levels remained basically consistent.

## 4. Discussion

This study examined the impact of wild and cultivated environments on the morphology, physiology, and metabolite profiles of *Rhodiola sachalinensis*. Our findings highlight significant environmental effects on plant morphology, antioxidant components, and secondary metabolite accumulation.

Previous studies comparing components between wild and cultivated environments, including those based on metabolomics, have revealed diverse insights across various plants. For instance, Mangoale et al. examined the chemical composition and antioxidant activity of *Alepidea amatymbica* Eckl. & Zeyh. from both wild and cultivated environments [38]. Compared the nutritional value and chemical composition of wild and cultivated *Portulaca oleracea* L. [39]. In terms of metabolomics, some researchers conducted metabolomics studies on salt stress in wild and cultured barley [40]. The changes in active ingredients of plants, including changes in metabolites in metabolomics, are intuitive manifestations of the adaptation of wild and cultivated species to different growth environments.

However, despite advancements in various analytical methods, morphological analysis remains the most direct and effective approach for exploring plant diversity [29,41]. Morphological changes not only serve as indicators of alterations in physiological metabolism but also manifest as outcomes of plant adaptation to the environment. Through the morphological observation of *R. sachalinensis*, it is evident that the wild type exhibits longer roots compared to the cultivated type, while the variation in aboveground parts is not notably significant. Wild *R. sachalinensis* showed longer roots, likely an adaptive trait for survival in rocky, high-altitude conditions. In contrast, cultivated plants had shorter roots, possibly due to nutrient-rich soil reducing the need for extensive root systems. This aligns with previous research [42], which noted shorter root systems in nutrient-abundant environments.

The synthesis of flavonoids in plants serves as a crucial mechanism to counterbalance excessive reactive oxygen species (ROS) production [43]. Through our investigations, it was observed that the wild-type *R. sachalinensis* consistently exhibits robust antioxidant properties, as evidenced by total phenols, total flavonoids, and antioxidant activity. The wild plants had higher levels of total phenols, flavonoids, and antioxidant activity than cultivated plants, suggesting that environmental stress may drive the production of antioxidant compounds to counter high UV exposure and low temperatures. Interestingly, phenolic and flavonoid content was higher in wild roots but lower in aerial parts compared to cultivated plants, indicating varied responses to environmental pressures in different plant parts.

The dynamics of soluble sugars and soluble proteins, critical for plant adaptation, are intricately linked to environmental conditions and span the entirety of the life cycle [44]. Adverse conditions such as variations in light, moisture, and temperature can result in a substantial reduction in sugar and protein levels [45]. Cultivated plants showed higher levels of soluble sugars and proteins, likely due to their stable growth environment. This pattern is important as these compounds act as signaling molecules under abiotic stress, playing crucial roles in low-temperature and salt stress responses. Notably, both in the roots and aboveground parts, the cultivated type exhibits higher levels of soluble sugars and soluble proteins than the wild type. This suggests that cultivated *R. sachalinensis* can accumulate more soluble sugars and proteins during its growth, potentially serving as a reservoir for nutritional resources. Conversely, the wild type may experience higher utilization of soluble sugars and proteins as a strategy to cope with adverse environmental conditions.

The changes in metabolites provide a molecular-level analysis of the plant’s survival status [46]. The variations in metabolites are particularly informative, illustrating the intuitive differences between wild and cultivated types in different environments. These changes in compounds directly impact the content of medicinal components. Wild *R. sachalinensis* roots contained significantly higher levels of key medicinal compounds, such as salidroside and tyrosol, likely reflecting a survival strategy under harsh conditions. Similar patterns have been observed in other medicinal plants like wild *Portulaca oleracea*, which also show higher antioxidant activity and nutrient content [47].

We have observed intriguing questions regarding the significant role played by certain parts in environments with poor nutrient supply, generating substances to resist harsh conditions. Simultaneously, we need to assess whether, in the current context of decreasing wild sources, cultivated environments can meet the demand for medicinal components. It is also crucial to explore methods for narrowing the differences between wild and cultivated sources. These are all issues worthy of further investigation.

## 5. Conclusions

We investigated the morphological characteristics of wild and cultivated sources of *R. sachalinensis*. We measured soluble sugars, soluble proteins, antioxidant activity, total phenols, total flavonoids, primary metabolites, and secondary metabolites in both sources. From a morphological perspective, we observed that wild *R. sachalinensis* possesses relatively longer roots. In terms of antioxidant capacity, the wild *R. sachalinensis* exhibited superior performance, closely associated with higher total phenols and total flavonoid content. Conversely, cultivated *R. sachalinensis* demonstrated advantages in soluble sugar and soluble protein content, indicating adaptation to current environmental conditions. Moreover, our analysis identified some significant differential metabolites, suggesting distinct metabolic processes between wild and cultivated environments during environmental changes.

## Data Availability

The original contributions presented in this study are included in the article/Supplementary Material. Further inquiries can be directed to the corresponding author.

## Supplementary Materials

The following supporting information can be downloaded at: https://www.mdpi.com/article/10.3390/plants13243544/s1.
