# Supplementary material for: Influence of Wild and Cultivated Environments on the Antioxidant and Medicinal Components of Rhodiola sachalinensis A. Boriss
Source: Plants (Basel). 2024 Dec 19;13(24):3544. doi: 10.3390/plants13243544 (PMC11680074; doi:10.3390/plants13243544)
Supplement: Supplementary file 1 [file plants-13-03544-s001.zip › plants-3361888-supplementary.pdf]

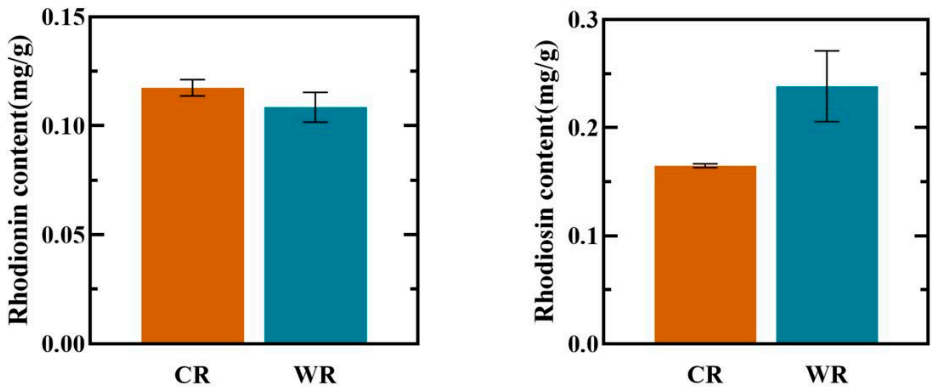

Figure S1 The content of rhodionin and rhodiosin in wild-type *Rhodiola sachalinensis* and cultivated *Rhodiola sachalinensis* varies.

Table S1 HPLC Gradient Elution Program

Time (min)	Solvent A (Water, %)	Solvent B (Acetonitrile, %)
0	90	10
15	75	25
30	60	40
45	30	70
48	90	10
60	90	10

Table S2 Metabolites in the leaves of *R. sachalinensis*

Index	VIP	P-value	FDR	Fold_Change	Log2FC	Type
Adipic acid	0.840114	0.42265	0.645009	4.35E-05	-14.4898	insig
Alanine	0.809297	0.429644	0.645009	0.220155	-2.18341	insig
Alizarin	0.474819	0.642864	0.793022	0.741607	-0.43127	insig
Aminobutanoic acid	0.986449	0.287332	0.645009	0.762606	-0.39099	insig
Aminocyclopentanecarboxylic acid	1.10298	0.260327	0.645009	4.579338	2.195139	insig
Aminopyrimidine	0.539047	0.598929	0.749771	1.654172	0.72611	insig
Androst	0.843255	0.42265	0.645009	0.000147	-12.7358	insig
Arabinitol	1.218729	0.227461	0.645009	0.047453	-4.39734	insig
Arabinofuranose	0.249884	0.813432	0.916466	0.836132	-0.2582	insig
Arabinose	0.835993	0.42265	0.645009	5.74E-05	-14.0884	insig
Benzaldehyde-para-carboxylic acid trimethylsilyl ester	1.311042	0.1958	0.645009	2498	11.28656	insig
Benzenamine	1.307283	0.191549	0.645009	0.000713	-10.4546	insig
Benzene	1.826559	0.013722	0.386492	0.000365	-11.4184	down
Benzenedicarboxylic acid	1.310685	0.189613	0.645009	2.00E-05	-15.6119	insig
Benzodiazepin	0.640027	0.513337	0.683482	2.357173	1.237058	insig
benzoic acid	0.111913	0.896487	0.971194	1.172887	0.230063	insig
Benzylquinoline	0.123349	0.915381	0.97911	1.132983	0.180127	insig
Butanal	0.739623	0.44899	0.645009	2.384731	1.253827	insig
Butanedioic acid	1.362047	0.16282	0.645009	2.38872	1.256238	insig
Butanediol	0.90569	0.395405	0.645009	8.911131	3.155608	insig
Butanoic acid	0.030082	0.991957	0.994802	1.011307	0.016221	insig
Butenedioic acid	1.19865	0.198728	0.645009	1.9238	0.943959	insig
Butenoic acid	0.012629	0.975112	0.994802	1.048048	0.067705	insig
Cadaverine	0.946091	0.352706	0.645009	0.425356	-1.23326	insig
Caffeic acid	1.145939	0.195038	0.645009	2.023849	1.017102	insig
carboxylic acid	0.843255	0.42265	0.645009	0.000194	-12.3287	insig
Cellobiose	1.077846	0.297639	0.645009	1.20E-06	-19.6719	insig
Citric acid	1.590517	0.040161	0.555633	2.549225	1.350059	up
Copper phthalocyanine	1.295384	0.198388	0.645009	0.000273	-11.8409	insig
Cyclohexadiene	0.840372	0.388451	0.645009	0.362669	-1.46328	insig
cyclohexane	0.857955	0.42265	0.645009	1222.333	10.25542	insig
cyclohexene	0.199018	0.830612	0.92351	0.839862	-0.25178	insig
Cyclopentadiene	0.020427	0.993055	0.994802	1.013173	0.018881	insig
Cyclopentene	0.841227	0.42265	0.645009	1323	10.3696	insig
cyclopropane	0.621041	0.524004	0.683482	2.700296	1.433117	insig
Danthron	0.682359	0.518191	0.683482	0.512589	-0.96413	insig
Decane	0.03836	0.970341	0.994802	1.047937	0.067553	insig
Dihydroxybutanoic acid tritms	1.268435	0.213219	0.645009	2079.667	11.02214	insig
Dimethyl-propyl 2,2-dimethyl-propanesulfinyl sulfone	0.060263	0.959919	0.994802	0.936249	-0.09504	insig
Dipentyl-heptabarbital	0.727152	0.486058	0.67331	0.150831	-2.72899	insig
Diphenyl isophthalate	1.467999	0.111374	0.645009	3.605821	1.850328	insig
Diphenyl-2-azafluorene	0.732022	0.450362	0.645009	0.370565	-1.4322	insig

Index	VIP	P-value	FDR	Fold_Change	Log2FC	Type
Disiloxane	0.020166	0.975968	0.994802	1.026184	0.03729	insig
Di-tert-butylphenoxytrimethylsilane	0.691578	0.502222	0.683482	1.410087	0.495784	insig
dl-2-Benzylaminooctanol	0.841227	0.42265	0.645009	2502.667	11.28925	insig
Dodecane	1.254988	0.149251	0.645009	4.167465	2.05917	insig
Erythritol	0.947649	0.363111	0.645009	0.036778	-4.76501	insig
Ethane	0.052087	0.970647	0.994802	0.946272	-0.07967	insig
ethanol	0.814094	0.405859	0.645009	0.29211	-1.77542	insig
Ethanolamine	0.115197	0.912778	0.97911	1.066081	0.092316	insig
Ethene	1.042504	0.314182	0.645009	0.278239	-1.8456	insig
Ethenediol	0.908822	0.384638	0.645009	0.050885	-4.29663	insig
Ethylene glycol	1.325058	0.183508	0.645009	8.33E-05	-13.5506	insig
Fluoro-2-methoxyphenol	0.274082	0.770232	0.886381	1.416277	0.502103	insig
Fructofuranose	1.242576	0.21688	0.645009	0.111868	-3.16013	insig
Fructose	1.839922	0.001466	0.123913	0.172333	-2.53673	down
Fumaric acid	0.835993	0.42265	0.645009	0.000205	-12.2488	insig
Galactaric acid	0.759234	0.438894	0.645009	2.387741	1.255647	insig
Galactitol	0.394426	0.705575	0.839733	0.555463	-0.84824	insig
Galactofuranoside	0.827652	0.427829	0.645009	0.131064	-2.93166	insig
Galactopyranose	0.840114	0.42265	0.645009	0.000132	-12.8846	insig
galactopyranoside	1.336867	0.179951	0.645009	0.230009	-2.12024	insig
Galactose	1.32503	0.183535	0.645009	2.77E-05	-15.1379	insig
Galactose oxime	1.726293	0.042741	0.555633	0.013105	-6.25371	down
Gallic acid	1.735569	0.035625	0.547328	4.362239	2.125069	up
Gentiobiose	0.843255	0.42265	0.645009	2.54E-05	-15.2651	insig
Glucitol	1.254738	0.214119	0.645009	12.73387	3.670599	insig
Gluconic acid	0.236804	0.800828	0.914459	0.751809	-0.41156	insig
glucopyranoside	0.626186	0.550807	0.7052	0.3244	-1.62415	insig
Glucose	0.941677	0.344178	0.645009	2.606859	1.382313	insig
Glucose oxime	0.841227	0.42265	0.645009	181626	17.47061	insig
Glucuronic acid	0.835993	0.42265	0.645009	6.01E-05	-14.0217	insig
Glyceric acid	1.691305	0.028417	0.48024	2.67242	1.418147	up
Glycerol	0.606893	0.54012	0.696797	1.18283	0.242243	insig
Glycine	0.857955	0.42265	0.645009	4274.667	12.0616	insig
Heptane	1.059795	0.297374	0.645009	2201.667	11.10438	insig
Heptylamine	1.316562	0.18714	0.645009	3.60E-05	-14.763	insig
hexane	0.433013	0.649962	0.795968	0.60743	-0.71921	insig
Hexanedioic acid	1.097132	0.251506	0.645009	0.208405	-2.26254	insig
Hydroxybenzoic acid	0.481667	0.626575	0.778612	1.690227	0.757217	insig
Hydroxybutanoic acid	0.418897	0.686992	0.823416	1.902817	0.928137	insig
Idopyranuronic acid	0.835993	0.42265	0.645009	8.34E-05	-13.5488	insig
Isoleucine	1.323802	0.185556	0.645009	8756.333	13.09611	insig
Isophthalic acid	0.847015	0.42511	0.645009	4.660054	2.220347	insig
L-5-Oxoproline	1.293948	0.192691	0.645009	47290.33	15.52926	insig
Lactic Acid	1.276868	0.156851	0.645009	1.457327	0.543324	insig
Lactose	0.913652	0.383125	0.645009	3.37E-06	-18.1773	insig

Index	VIP	P-value	FDR	Fold_Change	Log2FC	Type
Levoglucosan	0.841227	0.42265	0.645009	40234.67	15.29615	insig
Lyxofuranoside	1.289521	0.200425	0.645009	0.029835	-5.06683	insig
Lyxose	0.170573	0.864587	0.955002	0.822629	-0.28169	insig
Malic acid	1.497256	0.094675	0.645009	2.27623	1.186647	insig
Maltose	0.816799	0.435009	0.645009	0.070532	-3.82558	insig
Mannitol	0.620557	0.51603	0.683482	0.606906	-0.72045	insig
Mannobiose	1.390019	0.159644	0.645009	4.57E-07	-21.06	insig
Mannose	0.767069	0.44749	0.645009	0.385671	-1.37456	insig
methanol	0.289745	0.757509	0.88289	0.69735	-0.52005	insig
Methanone	0.840114	0.42265	0.645009	0.003827	-8.02975	insig
Methoxyamine	0.660821	0.525756	0.683482	1.630193	0.705043	insig
Methoxyethanol	0.82018	0.42265	0.645009	1570.667	10.61716	insig
Methoxyphenol	1.167856	0.244458	0.645009	2369.333	11.21027	insig
Methyl galactoside	1.069847	0.2947	0.645009	0.115733	-3.11113	insig
Methyl pentopyranoside	1.209998	0.227365	0.645009	0.084003	-3.57341	insig
Methyl xylopyranoside	0.835993	0.42265	0.645009	3.15E-05	-14.9548	insig
Methyl-6-hepten-4-olide	0.840114	0.42265	0.645009	0.001245	-9.64985	insig
Monopalmitin	1.307437	0.197243	0.645009	11697.67	13.51393	insig
Muco-Inositol	0.009199	0.994802	0.994802	1.00793	0.011395	insig
Myo-Inositol	0.829421	0.413853	0.645009	0.213653	-2.22666	insig
N-Methoxy-N-methylamino-N	0.554837	0.588624	0.742369	2.889423	1.530781	insig
Nonane	0.887417	0.342657	0.645009	1.368881	0.452997	insig
Norleucine	1.161963	0.250142	0.645009	2423.333	11.24278	insig
Norvaline	1.355935	0.162728	0.645009	9.649734	3.270489	insig
Palmitic Acid	0.169672	0.873373	0.957623	1.13311	0.180288	insig
Penicillamine	0.892917	0.390673	0.645009	0.010678	-6.54921	insig
Pentanedioic acid	0.763478	0.459921	0.653166	0.229463	-2.12367	insig
Pentaric acid	1.457599	0.133286	0.645009	0.009355	-6.74009	insig
Pentasiloxane	1.817449	0.011893	0.386492	0.19554	-2.35446	down
phenol	0.80366	0.441494	0.645009	0.047797	-4.38694	insig
phosphine	0.019804	0.994093	0.994802	0.988928	-0.01606	insig
Phosphoric acid	1.735349	0.021067	0.3956	0.133752	-2.90236	down
Phthalazine	1.161144	0.255082	0.645009	0.000327	-11.5763	insig
Phthalic acid	0.902472	0.397015	0.645009	91.86364	6.521422	insig
Pipecolic acid	0.857955	0.42265	0.645009	23157.67	14.4992	insig
Propanedioic acid	0.840114	0.42265	0.645009	0.000143	-12.769	insig
Propanoic acid	0.355098	0.735012	0.862618	0.574313	-0.80009	insig
Propenoic acid	0.845005	0.419693	0.645009	0.010581	-6.56232	insig
Propylacridine	1.859357	0.004934	0.208481	0.000106	-13.2065	down
Propylamine	0.883569	0.385141	0.645009	0.475711	-1.07184	insig
Propylene glycol	0.857955	0.42265	0.645009	2792.333	11.44726	insig
Putrescine	0.715377	0.470784	0.662008	3.801295	1.926491	insig
pyridine	0.354892	0.715654	0.845773	1.681626	0.749857	insig
Pyridinol	0.226964	0.828965	0.92351	0.866855	-0.20614	insig
Pyrimidinedione	0.414757	0.679936	0.82078	1.575356	0.655678	insig

Index	VIP	P-value	FDR	Fold_Change	Log2FC	Type
Pyrogallol	1.325867	0.183626	0.645009	1830.667	10.83815	insig
Quinoline	0.693612	0.496821	0.682625	2.641111	1.401145	insig
Quinolinecarboxylic acid	1.284744	0.203756	0.645009	2853	11.47826	insig
Rhamnose	1.22844	0.215493	0.645009	0.225639	-2.14791	insig
Ribitol	0.81655	0.438269	0.645009	0.039721	-4.65395	insig
ribofuranoside	0.857955	0.42265	0.645009	81478.67	16.31413	insig
Ribonic acid	1.2852	0.169242	0.645009	3.027329	1.598045	insig
Ribose	0.843255	0.42265	0.645009	1.26E-05	-16.2792	insig
Sedoheptulose	0.614402	0.564572	0.717388	1.634883	0.709187	insig
Serine	1.764958	0.020568	0.3956	2.311113	1.208588	up
Silane	0.744373	0.473982	0.662008	0.170606	-2.55126	insig
Silanol	1.100406	0.274624	0.645009	0.14748	-2.76141	insig
silatrane	0.669425	0.513505	0.683482	0.567835	-0.81646	insig
Sorbofuranose	0.218816	0.811712	0.916466	1.452342	0.538381	insig
Stearic acid	1.151461	0.261317	0.645009	1.809296	0.855429	insig
Succinic acid	0.439336	0.666873	0.810802	0.472018	-1.08309	insig
Sucrose	1.804528	0.019824	0.3956	0.010007	-6.64289	down
Sulfonyldiphthalic dianhydride	1.326702	0.183855	0.645009	12143.33	13.56788	insig
Sulfurous acid	0.136642	0.878294	0.957623	0.837624	-0.25563	insig
Talofuranose	0.843255	0.42265	0.645009	6.34E-05	-13.9442	insig
Terephthalic acid	0.887255	0.405586	0.645009	17.8632	4.158919	insig
tert-Butyl cyclopropylmethyl sulfoxide	0.050457	0.974017	0.994802	0.952381	-0.07039	insig
Tetramethylcyclopentene	0.954385	0.358764	0.645009	0.62863	-0.66972	insig
Tetrasiloxane	1.288572	0.182687	0.645009	0.336413	-1.5717	insig
Tetrazol	1.260806	0.142719	0.645009	0.4622	-1.11341	insig
Threitol	0.023473	0.977582	0.994802	0.964251	-0.05252	insig
Threonine	1.781383	0.003912	0.208481	5.476253	2.453189	up
Threose	0.840114	0.42265	0.645009	3.14E-05	-14.9593	insig
Trifluoromethylbenzylamine	1.322531	0.184485	0.645009	7.17E-05	-13.7686	insig
Trihydroxybutyric acid tetrakis	0.848347	0.370296	0.645009	0.577653	-0.79173	insig
Trisiloxane	0.291876	0.770994	0.886381	1.592904	0.67166	insig
Undecane	1.149246	0.260575	0.645009	0.000222	-12.1373	insig
urea	1.411805	0.149222	0.645009	2.374938	1.24789	insig
Valine	1.850476	0.000391	0.06612	8.588596	3.102422	up
Xylonic acid	0.857955	0.42265	0.645009	12895	13.65452	insig
Xylopyranose	0.835993	0.42265	0.645009	1.89E-05	-15.6913	insig
Xylose	1.10554	0.274188	0.645009	0.101805	-3.29612	insig

Table S3 Metabolites in the root of *R. sachalinensis*

	Index	VIP	P-value	FDR	Fold_Change	Log2FC	Type
1	Adipic acid	1.203734	0.301204	0.768477	4.67E-05	-14.3861	insig
2	Alanine	0.120874	0.910887	0.958828	0.900531	-0.15115	insig
3	Alizarin	1.977295	0.021749	0.489364	0.000572	-10.7709	down
4	Aminobutanoic acid	0.136637	0.887974	0.95463	0.962638	-0.05494	insig
5	Aminocyclopentanecarboxylic acid	0.976504	0.40826	0.768477	0.163694	-2.61093	insig
6	Aminopyrimidine	1.087017	0.36163	0.768477	2.172433	1.119312	insig
7	Androst	0.70036	0.543679	0.808767	0.725661	-0.46263	insig
8	Arabinitol	0.554362	0.60756	0.831877	0.393981	-1.3438	insig
9	Arabinofuranose	0.997105	0.368201	0.768477	0.169876	-2.55745	insig
10	Arabinose	0.237075	0.837885	0.936765	1.311523	0.391244	insig
11	Benzaldehyde-para-carboxylic acid trimethylsilyl ester	0.891018	0.42265	0.768477	0.001463	-9.41645	insig
12	Benzenamine	0.740512	0.48512	0.805924	2.843345	1.507589	insig
13	Benzene	0.307818	0.791212	0.918827	1.520078	0.604146	insig
14	Benzenedicarboxylic acid	0.891018	0.42265	0.768477	0.000508	-10.9435	insig
15	Benzenediol	0.478725	0.697801	0.878351	1.839526	0.879334	insig
16	Benzodiazepin	0.94975	0.42265	0.768477	1956.667	10.93418	insig
17	benzoic acid	0.94875	0.42265	0.768477	0.003584	-8.12412	insig
18	Benzylquinoline	0.891018	0.42265	0.768477	0.00019	-12.3621	insig
19	Butanal	0.10781	0.944699	0.971691	1.069582	0.097047	insig
20	Butanedioic acid	0.79461	0.426932	0.768477	1.402076	0.487564	insig
21	Butanediol	1.77668	0.045874	0.768477	0.321343	-1.63781	down
22	Butanoic acid	0.982593	0.392026	0.768477	0.149787	-2.73902	insig
23	Butenedioic acid	0.615539	0.566137	0.808767	0.314963	-1.66675	insig
24	Butenoic acid	0.892708	0.45477	0.794743	12.53014	3.647331	insig
25	Cadaverine	0.994364	0.398047	0.768477	3.332346	1.736538	insig
26	Caffeic acid	0.714402	0.539633	0.808767	0.258628	-1.95105	insig
27	carboxylic acid	0.572498	0.624748	0.831886	2.466102	1.302232	insig
28	Cellobiose	0.118236	0.899417	0.95463	1.067039	0.093613	insig
29	Citric acid	1.119295	0.313529	0.768477	0.334481	-1.58	insig
30	Copper phthalocyanine	0.243632	0.830229	0.936765	1.395091	0.48036	insig
31	Cyclohexadiene	0.95692	0.42265	0.768477	1505	10.55555	insig
32	cyclohexane	0.099863	0.931134	0.968489	1.139479	0.188375	insig
33	cyclohexene	0.924033	0.391012	0.768477	0.91202	-0.13286	insig
34	Cyclopentadiene	0.891018	0.42265	0.768477	0.00019	-12.3597	insig
35	Cyclopentene	0.187666	0.901595	0.95463	0.910856	-0.1347	insig
36	Decane	0.674422	0.51651	0.808767	1.391718	0.476867	insig
37	Dihydroxybutanoic acid tritms	0.94975	0.42265	0.768477	4343.333	12.08459	insig
38	Dimethyl-propyl 2,2-dimethyl-propanesulfinyl sulfone	1.220129	0.252063	0.768477	3.567121	1.83476	insig
39	Dipentyl-heptabarbital	1.507225	0.183578	0.768477	3849	11.91027	insig
40	Diphenyl isophthalate	0.351333	0.728802	0.886194	0.678752	-0.55904	insig
41	Diphenyl(tert-butyl)silyloxy-4-methoxybenzene	1.978614	0.020613	0.489364	1470.667	10.52225	up

	Index	VIP	P-value	FDR	Fold_Change	Log2FC	Type
42	Diphenyl-2-azafluorene	0.212599	0.879091	0.95463	0.875682	-0.19152	insig
43	Disiloxane	1.046232	0.374071	0.768477	7.492562	2.905459	insig
44	Di-tert-butylphenoxytrimethylsilane	1.2183	0.276448	0.768477	2.481974	1.311488	insig
45	dl-2-Benzylaminooctanol	0.416486	0.716212	0.883001	1.830666	0.872368	insig
46	Dodecane	0.007948	0.998952	0.998952	1.001978	0.002851	insig
47	Epigallocatechin	2.075049	0.000618	0.037078	26645	14.70158	up
48	Erythritol	0.879871	0.42265	0.768477	4186	12.03136	insig
49	Ethane	0.891018	0.42265	0.768477	0.003337	-8.22721	insig
50	ethanol	0.124807	0.889584	0.95463	0.834657	-0.26074	insig
51	Ethanolamine	0.409535	0.674213	0.872863	0.767346	-0.38205	insig
52	Ethene	1.33268	0.237488	0.768477	0.000599	-10.7048	insig
53	Ethenediol	0.683644	0.4993	0.805924	0.424852	-1.23497	insig
54	Ethylamine	0.77326	0.476114	0.805924	0.459324	-1.12242	insig
55	Ethylene glycol	1.060902	0.339708	0.768477	3.65E-05	-14.7424	insig
56	Fluoro-2-methoxyphenol	0.158639	0.873012	0.95463	0.905681	-0.14293	insig
57	Fructofuranose	0.403847	0.715589	0.883001	0.8809	-0.18295	insig
58	Fructose	1.010713	0.389852	0.768477	2.974957	1.572869	insig
59	Fumaric acid	1.45164	0.183656	0.768477	0.000302	-11.6913	insig
60	Galactaric acid	1.382458	0.216934	0.768477	9275	13.17913	insig
61	Galactitol	0.704059	0.51689	0.808767	4.577511	2.194563	insig
62	Galactofuranoside	0.879871	0.42265	0.768477	2012.333	10.97465	insig
63	Galactopyranose	2.057724	0.000335	0.030133	0.059346	-4.0747	down
64	galactopyranoside	0.792569	0.48151	0.805924	0.566836	-0.819	insig
65	Galactopyranoside	1.115691	0.302994	0.768477	0.19978	-2.32352	insig
66	Galactose	0.818027	0.48824	0.805924	6.433934	2.685701	insig
67	Galactose oxime	1.629929	0.123747	0.768477	1.611511	0.688414	insig
68	Gallic acid	0.3131	0.78805	0.918827	1.175323	0.233057	insig
69	Gentiobiose	1.970257	0.009059	0.326137	0.17987	-2.47498	down
70	Glucitol	0.88118	0.425932	0.768477	3.464009	1.792443	insig
71	Gluconic acid	0.148879	0.924965	0.967987	0.900777	-0.15076	insig
72	glucopyranoside	0.236224	0.85211	0.946788	0.813623	-0.29757	insig
73	Glucose	0.457566	0.706991	0.883001	0.628144	-0.67083	insig
74	Glucose oxime	0.216152	0.811984	0.930937	0.689	-0.53742	insig
75	Glucuronic acid	1.496709	0.184194	0.768477	3.20E-05	-14.9328	insig
76	Glyceric acid	1.642044	0.101431	0.768477	3.123658	1.643236	insig
77	Glycerol	1.413859	0.217256	0.768477	3.640321	1.864066	insig
78	Glycine	0.770331	0.485752	0.805924	0.465376	-1.10353	insig
79	Heptane	0.429995	0.696298	0.878351	0.578459	-0.78971	insig
80	Heptylamine	0.903796	0.449337	0.792947	14.95098	3.902168	insig
81	hexane	0.396787	0.728202	0.886194	1.773029	0.826216	insig
82	Hydroxybenzoic acid	1.451264	0.184067	0.768477	0.00029	-11.7538	insig
83	Hydroxybutanoic acid	0.879871	0.42265	0.768477	2956.667	11.52976	insig
84	Idopyranuronic acid	1.02769	0.33762	0.768477	0.332536	-1.58842	insig
85	Isoleucine	0.946773	0.42265	0.768477	0.000839	-10.2196	insig
86	Isophthalic acid	0.010959	0.952606	0.972622	0.928025	-0.10777	insig

	Index	VIP	P-value	FDR	Fold_Change	Log2FC	Type
87	Isoquinolinium	0.637475	0.558598	0.808767	0.627055	-0.67334	insig
88	L-5-Oxoproline	0.73144	0.532421	0.808767	4.083013	2.029634	insig
89	Lactic Acid	1.656304	0.076276	0.768477	2.83436	1.503023	insig
90	Lactose	1.46709	0.143952	0.768477	0.509818	-0.97195	insig
91	Leucine	0.080258	0.978834	0.989832	0.962476	-0.05518	insig
92	Levoglucosan	0.539245	0.595152	0.831877	1.77104	0.824597	insig
93	Lyxofuranoside	0.466476	0.657375	0.857446	1.307004	0.386264	insig
94	Lyxose	1.402961	0.197884	0.768477	33447.33	15.0296	insig
95	Malic acid	0.073647	0.956412	0.972622	0.98676	-0.01923	insig
96	Maltose	1.275421	0.201276	0.768477	1.98427	0.988609	insig
97	Mannitol	0.505038	0.678894	0.872863	0.799621	-0.32261	insig
98	Mannobiose	1.224893	0.278605	0.768477	0.193038	-2.37304	insig
99	Mannose	0.31679	0.80901	0.930937	1.227428	0.295638	insig
100	Melibiose	1.57877	0.142912	0.768477	106133.3	16.69552	insig
101	Methanone	0.53802	0.614664	0.831877	0.402766	-1.31199	insig
102	Methoxyamine	0.996654	0.346029	0.768477	2.924285	1.548084	insig
103	Methoxyethanol	1.453674	0.183574	0.768477	0.000252	-11.9556	insig
104	Methoxyphenol	0.599343	0.609274	0.831877	0.61143	-0.70974	insig
105	Methyl galactoside	1.196051	0.30677	0.768477	250070.7	17.93198	insig
106	Methyl pentopyranoside	1.360949	0.223401	0.768477	0.094572	-3.40245	insig
107	Methyl xylopyranoside	0.669854	0.551084	0.808767	0.603528	-0.72851	insig
108	Methyl-6-hepten-4-olide	0.682013	0.501464	0.805924	2.455357	1.295933	insig
109	Methylbenzothiophene	1.031427	0.375315	0.768477	0.214065	-2.22388	insig
110	Monopalmitin	0.513835	0.628536	0.831886	1.471408	0.557197	insig
111	Muco-Inositol	0.94875	0.42265	0.768477	3.74E-05	-14.7054	insig
112	Myo-Inositol	1.218632	0.287473	0.768477	93824	16.51767	insig
113	naphthalene	1.337729	0.220321	0.768477	29.90314	4.902225	insig
114	N-Methoxy-N-methylamino-N	0.446841	0.696293	0.878351	1.935587	0.952771	insig
115	Nonane	1.006644	0.343936	0.768477	0.349683	-1.51588	insig
116	Norleucine	0.891018	0.42265	0.768477	0.000504	-10.9532	insig
117	Norvaline	1.262687	0.275199	0.768477	0.000147	-12.7295	insig
118	Palmitic Acid	0.129996	0.882044	0.95463	1.023609	0.033665	insig
119	Penicillamine	0.95692	0.42265	0.768477	809.6667	9.661184	insig
120	Pentanedioic acid	0.318068	0.772268	0.914528	0.799686	-0.32249	insig
121	Pentanoic acid	1.139318	0.282055	0.768477	0.192413	-2.37772	insig
122	Pentaric acid	2.04764	0.000246	0.030133	0.181407	-2.46269	down
123	Pentasiloxane	1.091945	0.329013	0.768477	1.683796	0.751717	insig
124	Pentenoic acid	1.191828	0.28163	0.768477	0.000276	-11.8224	insig
125	phenol	1.986923	0.016322	0.489364	0.035757	-4.80562	down
126	phosphine	0.94975	0.42265	0.768477	904.3333	9.820711	insig
127	Phosphoric acid	0.570204	0.624424	0.831886	1.528939	0.61253	insig
128	Phthalazine	0.94975	0.42265	0.768477	124.3333	6.958069	insig
129	Phthalic acid	0.94875	0.42265	0.768477	0.001295	-9.59308	insig
130	Pipecolic acid	1.173733	0.259126	0.768477	0.224877	-2.15279	insig
131	propane	1.001847	0.341996	0.768477	2.960844	1.566008	insig

	Index	VIP	P-value	FDR	Fold_Change	Log2FC	Type
132	Propanedioic acid	0.946773	0.42265	0.768477	0.000514	-10.9268	insig
133	Propanoic acid	1.102845	0.346913	0.768477	2369.667	11.21047	insig
134	Propenoic acid	0.757469	0.525524	0.808767	4.259176	2.090574	insig
135	Propylacridine	0.891018	0.42265	0.768477	0.000411	-11.2493	insig
136	Propylamine	1.745208	0.044685	0.768477	4.688491	2.229124	up
137	Propylene glycol	1.229059	0.28601	0.768477	1565.667	10.61256	insig
138	Putrescine	1.242673	0.276321	0.768477	12659	13.62788	insig
139	pyridine	0.619854	0.584496	0.82842	1.648968	0.721564	insig
140	Pyridinol	0.858628	0.444296	0.791814	1.297523	0.37576	insig
141	Pyrimidinedione	0.946773	0.42265	0.768477	0.003352	-8.22078	insig
142	Pyrogallol	0.541004	0.604797	0.831877	0.563968	-0.82632	insig
143	Quinoline	0.581456	0.614091	0.831877	0.388186	-1.36518	insig
144	Quinolinecarboxylic acid	0.093118	0.936206	0.968489	0.886151	-0.17438	insig
145	Rhamnose	1.144406	0.318428	0.768477	4.470861	2.160553	insig
146	Ribitol	0.04368	0.997994	0.998952	0.996225	-0.00546	insig
147	Ribofuranose	1.208755	0.284451	0.768477	0.213188	-2.2298	insig
148	ribofuranoside	1.10505	0.285116	0.768477	0.251086	-1.99374	insig
149	Ribonic acid	0.94975	0.42265	0.768477	8028	12.97082	insig
150	Ribose	0.802347	0.494492	0.805924	0.168782	-2.56677	insig
151	Sedoheptulose	0.984417	0.372789	0.768477	0.129767	-2.946	insig
152	Serine	0.282135	0.76066	0.906747	0.786386	-0.34669	insig
153	Silane	0.707079	0.494794	0.805924	0.525429	-0.92843	insig
154	Silanol	1.792986	0.073891	0.768477	5.094692	2.348995	insig
155	silatrane	1.042356	0.369171	0.768477	3.516465	1.814126	insig
156	Sorbofuranose	0.95692	0.42265	0.768477	110030.7	16.74755	insig
157	Sorbopyranose	0.665711	0.564045	0.808767	1.385968	0.470894	insig
158	Stearic acid	0.614694	0.559364	0.808767	0.935008	-0.09695	insig
159	Succinic acid	0.94975	0.42265	0.768477	4932.333	12.26805	insig
160	Sucrose	0.704888	0.521347	0.808767	0.603136	-0.72945	insig
161	Sulfonyldiphthalic dianhydride	0.94975	0.42265	0.768477	7835	12.93572	insig
162	Sulfurous acid	0.354016	0.760528	0.906747	1.63354	0.708002	insig
163	Talofuranose	0.17806	0.891403	0.95463	0.837063	-0.25659	insig
164	Terephthalic acid	0.992766	0.398364	0.768477	5.41E-05	-14.1731	insig
165	tert-Butyl cyclopropylmethyl sulfoxide	0.95692	0.42265	0.768477	105.6667	6.723377	insig
166	Tetramethylcyclopentene	1.397833	0.155383	0.768477	0.814588	-0.29586	insig
167	Tetrasiloxane	0.618866	0.548919	0.808767	1.904094	0.929105	insig
168	Tetrazol	0.946773	0.42265	0.768477	0.005357	-7.54432	insig
169	Threitol	0.964371	0.413764	0.768477	9.12E-06	-16.7432	insig
170	Threonine	0.28804	0.833763	0.936765	1.247685	0.319253	insig
171	Threose	0.557251	0.633724	0.83263	2.382494	1.252473	insig
172	Trifluoromethylbenzylamine	0.299864	0.783228	0.918827	0.646212	-0.62992	insig
173	Trihydroxybutyric acid tetrakis	1.226719	0.239868	0.768477	0.526539	-0.92539	insig
174	Trisiloxane	1.771076	0.084683	0.768477	0.243429	-2.03843	insig
175	Undecane	0.241103	0.832837	0.936765	1.387593	0.472585	insig
176	urea	0.893601	0.404926	0.768477	0.769082	-0.37879	insig

	Index	VIP	P-value	FDR	Fold_Change	Log2FC	Type
177	Valine	0.323189	0.733572	0.886194	0.744073	-0.42648	insig
178	Xylonic acid	2.048906	0.002523	0.113548	0.069276	-3.8515	down
179	Xylopyranose	0.688635	0.559025	0.808767	3.399408	1.765284	insig
180	Xylose	1.687751	0.071259	0.768477	2.078902	1.055822	insig
